# Learning *from* others is good, *with* others is better: the role of social interaction in human acquisition of new knowledge

**DOI:** 10.1098/rstb.2021.0357

**Published:** 2023-02-13

**Authors:** Sara De Felice, Antonia F. de C. Hamilton, Marta Ponari, Gabriella Vigliocco

**Affiliations:** ^1^ Institute of Cognitive Neuroscience, University College London (UCL), 17–19 Alexandra House Queen Square, London WC1N 3AZ, UK; ^2^ Experimental Psychology, 26 Bedford Way, London WC1H 0AP, UK; ^3^ School of Psychology, University of Kent, Canterbury CT2 7NP, UK

**Keywords:** social learning, social interaction, interactive learning, hyperscanning, ecological neuroscience, two-person neuroscience

## Abstract

Learning in humans is highly embedded in social interaction: since the very early stages of our lives, we form memories and acquire knowledge about the world from and with others. Yet, within cognitive science and neuroscience, human learning is mainly studied in isolation. The focus of past research in learning has been either exclusively on the learner or (less often) on the teacher, with the primary aim of determining developmental trajectories and/or effective teaching techniques. In fact, social interaction has rarely been explicitly taken as a variable of interest, despite being the medium through which learning occurs, especially in development, but also in adulthood. Here, we review behavioural and neuroimaging research on social human learning, specifically focusing on cognitive models of how we acquire semantic knowledge from and with others, and include both developmental as well as adult work. We then identify potential cognitive mechanisms that support social learning, and their neural correlates. The aim is to outline key new directions for experiments investigating how knowledge is acquired in its ecological niche, i.e. socially, within the framework of the two-person neuroscience approach.

This article is part of the theme issue ‘Concepts in interaction: social engagement and inner experiences’.

## 1. Introduction

Throughout our life, we acquire new information and form new conceptual representations largely in social contexts: for example, babies learn from their carers at home, pupils learn from teachers at school and by sharing their experiences with other students. In the same way, adult learning typically occurs in social contexts and in relation to peers, colleagues at work and/or mentors. Researchers in anthropology and sociology (e.g. [1,2]), as well as in developmental psychology (e.g. [3–7]) have emphasized in their work the importance of social interaction for learning and for development. However, cognitive psychology and neuroscience have traditionally studied cognition at the individual level. The ‘single-brain’ approach [8] studies brain and cognition using experimental designs involving a sample of participants (children or adults) completing a given task individually, and then makes inferences about how the brain works more generally. It is only in the past decade that cognitive neuroscientists have begun to move to a ‘second-person neuroscience' approach [9], which studies cognitive processes in interaction, including the back-and-forth dynamics between two or more people.

A significant component of human learning lies in the ability to *act on* and *interact with* the surrounding environment [10]. For example, during childhood, we learn to encode features of objects via manipulation (action), and we understand the space around us by mapping regions where objects are within or outside manual reach [11]. Previous literature has focused on solitary individuals performing actions (e.g. object manipulation) and showed how action supports learning (e.g. [12]). Here, we discuss the notion and present evidence showing that (solitary) action *per se* may not be enough, but rather that *action in interaction* may be key to support human learning, as exercised via many forms including gestures, object manipulation and language [13–16].

Here, we apply these ideas to the case of learning new concepts and knowledge. Our discussion is limited in scope as it considers especially cognitive neuroscience studies of learning. We primarily focus on conceptual learning, broadly defined (i.e. long-term memory for knowledge), but we extend the review discussion to other forms (e.g. single words, motor learning) when relevant. We first set out the theoretical framework and rationale of this review, providing some definitions and introducing some ideas relevant to the study of human learning. We then move to review the evidence in children and adults showing that learning benefits from social interaction across the lifespan. We also present neuroimaging studies to identify the neural signature of social interactive learning. In our discussion, we identify the possible cognitive mechanisms subserving interactive learning. We conclude by highlighting some methodological and theoretical issues and pose questions for future research.

### (a) Theories and definitions

**Figure 1.**
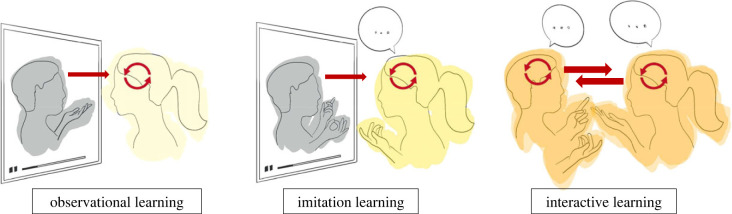
Schematic of three types of social learning. Arrows indicate information flow between teacher and learner in three types of social learning. From left to right: Observational learning: learner attends to information that flows from teacher to learner. Imitation learning: learner attends to information that flows from teacher to learner and repeats/imitates the teacher. Interactive learning: teacher and learner engage in social interaction and exchange reciprocal social signals. Importantly, information flows back and forth from teacher to learner. (Online version in colour.)

Human learning refers to any form of acquisition of new knowledge and skills by an individual. One can learn new information alone, e.g. memorizing events via reading a history book. However, often learning occurs with and from other people. When such learning occurs via transmission of information across members of a social group, it is defined as ‘social learning' [17]. Importantly, there are many ways in which learning can be social, depending on the role that the social agent(s) has in the learning process of a given individual. Therefore, the term social learning is a broad term that refers to any form of learning—such as motor, verbal and knowledge-based learning—via any form of social context, including observation of others [18,19], imitation [20] and interactive learning (figure 1).

*Observational learning* refers to learning via attending to someone else's actions and/or listening to them delivering information. *Imitation* refers to copying someone else [20]. Observational learning and imitation differ as observational learning only requires attention to the teacher, without immediate replication of their actions/words, while imitation learning involves observation plus active performance. In other words, imitational learning is defined by *action*. However, both observational and imitational learning can arise when a person watches a video of another person's actions or speech, with *no* interaction between the watcher and the video, and thus both involve the one-way transmission of information from teacher to student, where the learner is confined to the role of a receiver. By contrast, in *interactive learning*, *action occurs in interaction:* both the teacher and the student are concurrently engaged in the learning process and they can both take full turns during the interaction. While interactive learning can vary in terms of how interactive any given context is [21,22], with variations even within single episodes (e.g. sessions), by definition *any* given interaction draws in all agents as contributors: all can participate to the interaction in both explicit (e.g. verbal feedback) and implicit (e.g. body language) forms.

The impact of social learning for development has been largely acknowledged within sociocultural contexts, especially with reference to the collaborative nature of learning in interaction [23]. Within cognitive models, interactive learning is defined by a two-way exchange of signals that includes subtle but critical reciprocity from student to teacher (figure 1). These could indicate understanding (or lack thereof) as well as attentiveness (or inattentiveness) and thus allow the teacher to tune their lesson to the student. Here, we refer to reciprocity as any reaction fed-back during an online exchange that would inform the interlocutor(s) about the quality of the exchange (e.g. nodding for understanding, frowning for confusion etc.), and thus possibly allow for a (re)direction of behaviour(s), as well as opportunities for the learner to elaborate what is being discussed. Thus, learning in interaction requires mutual feedback between a *student* (or *learner*, who is acquiring new knowledge) and a *teacher* (who is providing new information). Importantly, independently of the type of social context, for (social) learning to occur there must be an enduring change in the learner's action and/or knowledge as a consequence of either observing, imitating or interacting with others [24].

Such categories have been developed over the past decades especially in the context of action/motor learning, and their applicability to knowledge-based learning may be less obvious, although still useful to draw some conceptual distinctions. We employ these categories here to draw the distinction between learning *from* others (imitation and observational learning) and learning *with* others (interactive learning). There are only few studies that directly contrast different types of learning. Examples include comparisons of physical or observational learning [25] and comparisons of sequence learning from imitation or verbal instruction [26] or of observational versus interactive learning [27,28].

Pioneering work from developmental psychologist Vygotsky [7,29] had long argued for a key role for the environment, especially social environment, in learning and development. His sociocultural theory of cognitive development views conceptual learning as an intrinsic social process. A number of other researchers in developmental psychology have also emphasized the importance of social interaction in cognitive and linguistic development (e.g. [30]). It is the case, however, that learner–teacher (or learner–learner) social dynamics has been largely neglected by modern cognitive neuroscience research, especially in adulthood. Methods adopted to study human learning have often included single-user tasks, where participants were required to memorize things from cards/screens, in very repetitive and highly constrained experimental paradigms (e.g. [31,32]). More recently, there has been a trend towards studying human learning in more dynamic social contexts (e.g. [33,34]). Despite the creditable effort to move away from the traditional reductionist approach [35], this new line of research has only partially included the social aspect in the study of human learning. Namely, at best social context has been included in the study design and data collection, while the focus of data analysis has been almost exclusively either on the *learner* [36] or (less often) on the *teacher* [37], and only rarely on the *interaction* [38]. This is also the case in many developmental studies that see the teacher (carer) as providing an input to the learner (the child; e.g. [39]). The problem with this is that conclusions may be based on a partial view of what is happening during a real-world student–teacher (or child–carer) interaction.

We propose to look at human social learning by considering three main elements from study design to data analysis and interpretation of results: the *learner*, the *teacher* and the *interaction* among the social agents involved in the learning processes (e.g. learner–teacher and/or learner–learner). We believe this approach will provide a systematic and comprehensive method to understand human learning in social contexts, by identifying the mechanisms subserving learning within individual cognitive and brain systems and as an interactive process. Importantly, we do *not* suggest that learning can *only* occur socially, rather that interactive learning is a qualitatively different phenomenon and involves different cognitive and physiological mechanisms from learning alone. As such, interactive learning must be studied taking social context into careful consideration and within clear definitions.

## 2. Review of the evidence

### (a) Human learning is social

#### (i) Social interaction is crucial for optimal development

Social interaction is crucial for optimal cognitive and brain development [40–42]. This statement is relatively uncontroversial and is supported by a large body of literature (for the latest systematic review on the topic see [43]). Taking together the results from their review, Ilyka and colleagues concluded that an optimal development of cognitive functions—as measured via heterogeneous neuropsychological test batteries—and brain—as measured via structural and functional analysis of selected regions and networks—is contingent on child–carer interaction during the child's first years of life. Out of 55 relevant publications included in their systematic review, only six looked at both child and carer and how the dyadic interaction impacted on cognitive and brain development [44–49]. Results coming from such an approach point to the importance of ‘sensitivity' and ‘reciprocity' of *both* agents for optimal child development, and in turn at the quality of the overall *interaction* to support cognition later in life.

Considering interactive learning specifically, the majority of work on children comes from studies on language acquisition: these robustly and consistently show that social interaction is a critical and constraining factor for successful language development [41]. In a pioneering study, Kuhl *et al*. [50] trained nine-month-old American babies to distinguish Chinese Mandarin sounds in three different conditions: in interaction with a native speaker, or by exposure to either videos or sound recordings from the same native speaker. Despite equivalent exposure time and content of Chinese sounds, only the group who engaged in live-interaction with the teacher showed learning, and being exposed to videos or sound recordings was associated with no learning. While this study is not strictly looking at knowledge-based learning (e.g. concepts), it provides strong evidence for the crucial role of interaction in children's learning over non-interactive learning methods.

More work on word acquisition during child–carer interaction has been conducted by Yu and Smith [51–54]. In their experimental paradigm, the infant and the carer engage in a series of free-play sessions during which they manipulate and name various objects (toys), while both wear head-movement sensors and eye-trackers. Crucially, in all their studies, the parent (teacher) is not aware that their infant's learning of the objects' names will be tested after the free-play session. This ensures that child–carer interactions are as natural as possible. By conducting a series of dyadic analyses, Yu & Smith [52] showed that 18-month-old infants were more likely to successfully learn objects' names if two things happened concurrently: (1) the infant (learner) held the object closer so that it was visually dominant within their visual field (over other competitor objects on the play table), and (2) the carer (teacher) named the object. Overall, these papers demonstrate the importance of social interaction in young children's word-learning.

The critical role of social interaction for optimal language development is relatively unsurprising, considering how heavily human language relies on the ‘social brain' and *vice versa* [41,55,56]. Also, in many developmental pathologies such as autism spectrum disorder, social cognition deficits and communicative disorders are co-occurring [57,58]. Given the highly interconnected nature of social cognition and language processing [59], learning language within a social context would be expected to be beneficial. Therefore, because of the strong relationship between communication and sociality, one may argue that the social-interaction advantage is limited to language development.

However, the beneficial effect of social interaction during development is not limited to language. Evidence from a variety of studies shows that social interaction supports learning more generally across different domains, including visuospatial categorization [60,61], procedural learning [62] and mathematical reasoning [34]. In their study, Kostyrka-Allchorne and colleagues [34] found that in a large group of 5-year-olds (*n* = 215), the physical presence of a teacher (versus having the teacher on screen) was associated with the highest learning, independent of whether children were observing the teacher playing with a shape or they were playing with it themselves. This study is a great example of why student–teacher interaction should be studied as interdependent: it is certainly unlikely that the mere presence of the teacher had something ‘magical' about it so that the child learned more when the teacher was physically there. Equally, audience effects—defined as ‘a change in behaviour caused by being observed by another person' ([63], pp. 160)—cannot explain the results, as students were explicitly observed in the on-screen condition too. Rather, there may well be something within the dynamic of learner–teacher interaction as it occurs face-to-face that positively impacted learning. For example, Marsh *et al.* [64] found that children showed more overimitation—i.e. unnecessarily copying actions of others—when an adult was demonstrating goal-oriented actions, compared to when demonstration was presented through a recorded video. This suggests that social factors may directly give rise to different behaviours (e.g. overimitation) during learning *with* others, and this may increase with age as people think more about social norms ([65], see §3 below for a discussion of the mechanisms subserving interactive learning).

#### (ii) Social interaction is a booster in adult human learning

Studies on interactive learning predominantly focus on children and young people because they are considered as the typical learners. Little is known about social learning in adults, and even less in interactive learning specifically. There is some evidence suggesting that, similar to what is found in children, social interaction acts as a catalyst for learning in adults too [27,66]. Again similarly to the literature on children, the majority of studies on interactive learning in adults have considered the domain of language, the focus being on second language acquisition [33,66–68]. In their study, Jeong *et al*. [33] asked 36 Japanese adults to learn two sets of unknown Korean spoken words via either translation or videos depicting social situations. Words encoded from the social condition showed significantly higher accuracy rates and faster reaction times than words encoded from the translation condition, and the social-video learning condition was also associated with higher activity in the right temporal parietal junction, right hippocampus and motor areas, as measured with functional magnetic resonance imaging. In their review, Verga & Kotz [66] reported evidence for the importance of sociality in adult learning: specifically, learning a second language in interaction with another person significantly improves long-term retention of new vocabulary.

Considering domains other than language, studies on adults have mainly looked at the impact of social interaction in comparison to online/virtual learning environments. Results are less conclusive: the majority of studies found no difference in learning outcomes between teaching live versus teaching through recorded videos [69–74]. In their intervention study, Brokfeld *et al*. [69] divided 296 medical students into four groups, three of which received 41 4 h lessons live, while the last group watched videos of the same lessons. The group assigned to the video condition changed daily, so that all students saw both live and video lectures. The effectiveness of the teaching method was evaluated by looking at students' performance on 301 multiple-choice questions of the medical exam. Similar approaches were adopted by the other studies cited here, and all found that learning performance did not differ across teaching methods. Despite no difference in objective performance, all these studies found that there were some differences in subjective evaluation, with the majority of students preferring live lessons. However, these studies did not control for exposure time: recorded material could be replayed multiple times while the live session was only live once. Studies that controlled for content and exposure time across conditions found a significant improvement in learning of medical students during social interactive lectures compared to recorded tutorials [75,76].

The studies reviewed above did not directly control the social factor during learning. In a study from our group [27], we designed two repeated-measure yoked-control experiments and tested learning of over 50 adults during online sessions in different conditions designed to specifically test different social factors. People learned some facts about uncommon items (musical instruments, ancient objects, exotic food and animals) in interaction with a teacher (experimenter) and some other facts from videos of another participant attending to the previous experimental session. Results robustly showed that performance was better for items learned in live-interaction with the teacher, and such advantages remained a week later. In this study, *all* conditions were *social*: however, while in the recorded-video condition, the student took part in *observational* social learning (learning by watching a video of another teacher–student interaction), during live sessions the student directly engaged with the teacher (*interactive* social learning).

Overall, studies of interactive learning in adults yield similar results to those in children. However, scholars are less unified on the notion that social factors matter in adult learning, possibly because adult learning—specifically of new concepts and information—is generally under-studied compared to child learning, and social factors in adulthood may be less critical than during development. We presented studies showing that, when exposure time is controlled for, there is an advantage for social interactive learning in adults too, and this is seen across a variety of stimulus types, including foreign languages, motor skills and—central to this paper—conceptual knowledge.

### (b) The neural signature of interactive learning

Hyperscanning has become increasingly popular over the past decade because it has the advantage of measuring brain activity from more than one individual at the same time, meaning the social brain can be studied while people engage in social interaction rather than in isolation [77–80].

In a five-person electroencephalography (EEG) hyperscanning study, Davidesco *et al*. [81] simultaneously measured brain activity from four students and their teacher during a science class. They found that alpha-band (8–12 Hz) brain-to-brain synchrony (i.e. across individuals), but not intra-brain alpha synchrony (i.e. within individuals), significantly predicted students' learning, as measured via performance in an immediate and a delayed test a week after the class. Moreover, moment-to-moment variation in alpha-band brain-to-brain synchrony during the class specifically predicted what information was retained by the students a week later. Alpha frequency band is a well-established neural index of attention [82], which suggests that learning was better predicted by moments when students were attuned (or paying attention) to the teacher, *and concurrently* the teacher was attuned (or paying attention) to the students.

The same research group conducted another EEG hyperscanning study where brain activity was recorded from 12 high school seniors simultaneously over a semester [83]. Recording took place during students' regular biology class and was repeated over 11 sessions. Results showed that the degree to which brain activity was synchronized across students predicted student class engagement (quantified as student appreciation ratings of different teaching styles and student daily self-reported focus). In particular, they conducted a group-based neural coherence analysis to link student-to-group brain synchrony to different predictors. They found that student focus predicted student-to-group synchrony above and beyond teaching style, and also students who were more focused on a given day showed higher synchrony for that day.

Given the association between learner-to-group synchrony and class engagement [83] and the link between engagement, attentional processes and learning [84], Dikker's group extended their work to ask whether learner-to-group or learner-to-teacher neural synchrony predicts learner's content retention [85]. Using a similar real-world classroom scenario, biology class materials were presented in either videos or live lectures, and students completed a multiple-choice quiz after each class. Results showed that brain-to-brain synchrony was higher for video than for live lectures (as expected by greater similarity in low-level processes during watching of the same video content). However, for live lectures only, social closeness to the teacher was related to learner–teacher brain synchrony: in other words, when there was a contingent learner–teacher interaction, this was reflected in their brain activity. In addition, learning performance correlated with learner–teacher closeness, but not with learner–teacher brain synchrony.

Using functional near-infrared spectroscopy (fNIRS), Holper *et al.* [38] recorded prefrontal brain activity during the Socratic dialog simultaneously in seventeen teacher–student pairs. The Socratic dialog is a classical teaching model where the teacher encourages learning by interrogating the students via a set of structured questions. They found that learning—as measured by students' correct responses—was associated with higher correlation of student–teacher brain activity.

Similar findings were obtained by another group that also used fNIRS to measure brain activity from learner and teacher dyads during the acquisition of a music song [86]. They found that brain activity in the bilateral Inferior Frontal Cortex showed learner–teacher synchronization. This was specifically associated with moments when the learner was observing the teacher and when learning was more interactive (measured in terms of turn-taking). Importantly, learner–teacher brain synchronization could predict a student's performance on the learned song. The same research group conducted a further study to investigate the causal role of such synchronization in learning [87]. They used transcranial alternating current stimulation to induce (or disrupt) brain synchrony in different conditions and found that induced teacher–learner neural coupling facilitated motor coordination, which in turn was associated with enhanced learning of novel Chinese songs. This intriguing work hints at many further interesting questions, and it will be useful to see it replicated and extended.

Overall, these studies demonstrate that brain-to-brain synchrony can be measured during interactive learning and may correlate with learning performance either across sessions [83] or even across individual events [81,86]. However, the presence of a correlation does not necessarily reveal the causal mechanism behind the effect, and we consider possible cognitive processes in the next section.

## 3. Learning from and with others: what is special about interactive learning?

In the previous sections, we have reviewed evidence showing that social interaction plays a key role in human learning across the lifespan and in a variety of cognitive domains, and also that it has a distinctive neural signature in the brain. It remains unclear, however, what social and cognitive mechanisms enhance learning in an interactive context. In this section, we consider some of the possible mechanisms that have been suggested and how these might be studied, before moving on to the wider implications of the work.

### (a) Cognitive mechanisms of interactive learning

A number of *cognitive mechanisms* have been proposed to account for the advantage of interactive learning over non-interactive learning, including stimulus saliency [88], social arousal [89], internal motivation [90], sustained attention [51], audience effects [63], eye-contact and gaze [91], joint attention [92], common ground [93], attunement and shared intentionality [94] and mutual predictions within inter-agents dynamics [95]. These can be distinguished on the basis of whether they describe effects within one individual alone (e.g. the learner) or whether they describe the learner–teacher relational dynamic [96]. Here, we discuss these systematically and evaluate them in relation to interactive learning.

### (b) Individual-based mechanisms: learner- and teacher-based approaches

Individual-based mechanisms include stimulus saliency, social arousal, internal motivation and sustained attention. Stimulus saliency and social arousal have been proposed as possible explanations for the social learning advantage on the basis of the well-established effects that faces (and social stimuli more generally) are processed differently from other types of non-social stimuli [97], and that we get more aroused in social than non-social situations [98]. In other words, according to these accounts, interactive learning is not ‘special' because it is *social per se*, but rather because social contexts share some features that make encoding of information somehow more memorable for future recalls [33]. In line with this, it has been found that distinct neural patterns of activation are associated with encoding and retrieving information learned in social contexts [33,67].

In addition to external bottom–up influences, the internal motivation of the learner may be fundamental to direct sustained attention, which in turn is an essential pre-requisite of learning [84]. There is no doubt that engaging with the learning material, by attending and processing the target information, is a strong predictor of how well we may be doing on a follow-up test. Yu *et al.* [51] demonstrated that this may be a very early mechanism that we engage from a young age. They found that in nine-month-olds, infant sustained attention predicted the learning of new vocabulary above and beyond joint attention between infant and their carer.

However, these factors seem to be telling only part of the story, and specifically the part concerning the learner. For example, Kostyrka-Allchorne *et al*. [34] found that five-year-olds learned about atypical geometric shapes better when there was a teacher physically present in the room with them. The observed learning benefit may well reflect some degree of arousal given by the physical presence of the teacher. However, it cannot be excluded that the presence of the teacher improved learning via mechanisms of relational dynamics [99]. We simply cannot exclude either option, due to the way the study was designed and the fact that the focus of the analysis was limited to the learner.

In fact, the role played by the teacher is crucial in determining the learning outcomes, and yet it is often underrepresented in the learning literature. Social communicative signals (both verbal and non-verbal e.g. pointing, eye-gaze) are overtly employed by an expert (the teacher) to transfer information to a novice (the learner [100,101]). The teacher's communication is functional to achieve successful teaching (and in turn someone else's learning), and as such is explicitly adjusted to maintain the learner's attention and assist information transfer. The teacher's communicative actions are therefore the other fundamental aspects to consider in the study of human learning.

The fact that teachers can adjust their verbal and non-verbal behaviour to assist the learner has been demonstrated in the case of both children and adult learners. For example, in their study, Brand *et al*. [102] showed that carers deliberately modified both their language and their actions when sharing information about novel object properties with infants compared to adults (who presumably were not novice to those objects). Similarly, Vigliocco *et al*. [103] further showed that carers adapted their language and their actions when presenting unknown versus known objects to their 2–3-year-old children. Similar modification of action with pedagogical intentions has been demonstrated in adults. In three experiments, McEllin *et al*. [104] recorded movements of participants playing simple xylophone melodies either alone, for a learner watching them, or together with another participant. They found that movement velocity was altered specifically in the condition when participants were playing to demonstrate musical sequence to a novice, compared to when they were playing alone or with someone else who was expert in the melody.

This literature demonstrates the importance of considering the teacher as well as the learner when studying how humans learn in interaction. However, looking at one *or* the other may not be enough. Studies that only consider one side of the interaction may overlook the social dynamic unfolding during interpersonal communication, and in turn reach partial and/or inaccurate conclusions regarding the mechanisms of human interactive learning.

In our work on social learning online [27], we found that visual social cues (teacher—face and hands) impacted learning differently depending on whether learning was interactive (student engaged in a live lecture) or observational (student learned from a pre-recorded video of a previous session): in two experiments, we showed a strong interaction effect between social contingency (live versus recorded contrast) and social richness (whether the face of the teacher was visible versus when just their hands or a slide was presented instead). To our knowledge, this was the first study showing that rich social cues specifically improve interactive but not observational learning. Our results point at the possibility that different cognitive mechanisms may support interactive and observational learning. In other words, there may be qualitatively different processes that are involved when we learn *with* others compared to when we are learning *from* others.

### (c) For every learner, there is a teacher, and *vice versa*: interaction-based approaches

Real-time social interaction involves rich and complex behavioural dynamics, with bi-directional responses and input between two or more people [52,105–107]. Such a multifaceted phenomenon is unlikely to rely on a single cognitive mechanism but rather a number of cognitive processes, which may be absent in a non-interactive situation. During interactive learning, learner–teacher dynamics may be characterized by joint attention [99], common ground [93], shared intentionality [94] or all these processes together [108]. These mechanisms of attunement between two or more conversational partners may allow information to be shared more effectively, and in turn be advantageous in those situations when we learn socially [109,110].

One approach to examining the rich and complex dynamics of interpersonal interaction is to argue that social interaction is more than just a context for social cognitive processes, but in fact replaces individual mechanisms [111]. In such an enactive model, the inter-personal relational dynamics become autonomous from the single individual parts making up the interaction. This implies that traditional single-person models have little relevance to the two-person interaction, and that researchers need to find a new type of dynamic model to understand interaction at a more abstract level.

However, we argue for a more incremental approach, where social interaction is included as an additional necessary element in the study of human cognition. As such, interpersonal interactions can be integrated into—and understood by building on—models of the solo brain. For example, we know that learning a new concept from a video will involve processes of perception, language and memory that allow the learner to integrate the new information into their existing knowledge structures. Learning a concept in interaction is likely to engage the same processes *plus* additional cognitive systems (e.g. joint attention, common ground etc.), where the moment-by-moment coordination of gaze and speech allows these additional processes to function smoothly. Understanding what these additional processes are and how they relate to enactive models will be an important research area in the future.

There is evidence that the quality and quantity of social cues present in a given interaction substantially affect the communicative outcome of that interaction [39,62]. Rich visual cues may enable stronger attunement by providing more information about the interaction partner's gaze and mental states [112,113]. Alksne [114] looked at what features in teaching videos improved the quality of the lecture in a group of young adults; they found that speaking over the presentation and making eye-contact significantly improved student engagement, which in turn has been positively associated with learning outcomes [115].

The fact that we somehow use our body to achieve a better attunment with our intelocutor(s) during social communication has been recently well demonstrated by Fini *et al*. [116]. In their study, they asked adults to guess concrete and abstract concepts from some photos, while being in interaction with an avatar. The avatar moved following the previously recorded kinematics of a real actor's arm, from which human movement was implicated to the avatar. They found an association between sociality (as measured by motor imitation and motor synchrony between the participant and the avatar) and guessing of abstract concepts. They argued that greater motor imitation showed by the learner specifically during more difficult trials (abstract words) reflected a greater longing for help: participants would try to attune more to the avatar to receive more hints and support in the guessing task. This interpretation is in line with the argument that social attunement may be a way to support efficient information transfer across interlocutors [117].

However, by looking only at the student, this study does not tell us much about whether the direction of such synchrony is unidirectional (from learner to teacher) or rather bi-directional. For example, Davidesco *et al*. [81] found that while learner-to-learner brain synchrony was instantaneous, learner-to-teacher brain synchrony could best predict learning when adjusting for a temporal lag of approximately 200 ms. Specifically, student brain activity would ‘tune in' to brain activity of the teacher only after a short delay, suggesting a sequential, lagged transfer of information from teachers to students. This type of data shows that, to fully grasp the neural mechanisms of interactive learning, it may be insufficient to focus on one social agent alone: dyadic analysis may carry more interesting and comprehensive information about these complex dynamics.

### (d) Synchronization as a signature of social learning

A growing body of the literature is emerging showing that a signature of interactive learning may be a *bi-directional* synchrony during teacher–learner interaction (see §2 for a review of the literature on this). When A interacts with B, both A and B would share some processing linked to the experience they are both part of, while brain of A would process information about B and brain of B would process information about A. By looking at individual brain systems *as part of* an interaction, we can start to understand the full temporal and behavioural dynamics that are reflected into individual brain activity (of interactive agents). These patterns of bi-directional coordination can be interpreted within the framework of the mutual-prediction hypothesis [95,118,119]. This claims that, when interacting with others, we engage in social prediction all the time in order to anticipate other people's actions and mental states [107,120]. Furthermore, when two people are both engaged in mutual prediction, their brain states will correlate and thus the signals recorded from their brains will correlate, giving rise to interbrain synchrony. Thus, predictive mechanisms present in individual brains can give rise to a consistent cross-brain signal that may predict learning [38,81,83,85,86].

However, claiming that brain to brain coupling on its own can tell us something conclusive about the quality of the social interaction, and even further, about the learning mechanisms of teacher–student social exchange, is at best ambitious—if not misleading (see [121] and [118] for a discussion on this). In conjunction with studying interpersonal brain synchrony, it is critical to understand the coordination of actions and how that relates to shared knowledge states (see [122] for a comprehensive framework of neural synchrony and its behavioural references). This may be particularly useful when learning from or teaching to someone else. In the case of interactive learning, the co-creation of knowledge and understanding is functional to the learning process: ideally, the teacher would want to share information, and the learner would want to tune in to their teacher to receive and process that information, while both would remain sensitive to feedback coming from their interlocutor to adjust their behaviour accordingly. It has been proposed that the extent to which people synchronize may be a proxy of ongoing exchanges during human social interaction [123,124]: in other words, high brain-to-brain synchrony across social agents should reflect behavioural inter-personal dynamics. Possibly, the objective is that of reducing prediction errors and increasing affiliation and communicative benefits [125]. Therefore, integrating behavioural data into hyperscanning studies is necessary to achieve a more comprehensive and meaningful knowledge of how humans learn from and with others.

In fact, being a form of social interaction, good pedagogy would be therefore characterized by continuous reciprocity: the teacher would monitor the audience's engagement and understanding, and use the audience feedback to adapt their performance as needed. Such mutual prediction engages the brain in a constant probabilistic estimate of occurrence of external experiences based on expected outcome. These may be plausible mechanisms underlying inter-personal synchrony and shared neural representations typical of social situations [96]. Studies have shown that interpersonal synchrony manifests across multiple levels during social interaction, including motor coordination [126,127], action coordination and decision-making [128] and verbal coordination [129–131]. In addition, person-to-person synchrony has been reported even at the physiological [132,133] and neural levels [122,125,134,135].

The study of interactive learning cannot answer questions on how individual cognitive mechanisms work *per se*, unless research considers the individual agents alone *and as part of* an integrated social dynamic where they learn from (and/or with) one another. Taking a second-person neuroscience approach [9,118] across all stages of the experimental work is particularly important as we are moving away from studying learning in isolation to study learning in social contexts: we must study interactive minds as they are found in the real world in the context of rich interactions, to fully understand interactive learning dynamics as they unfold [136]. We believe that this approach can give us a comprehensive understanding of what factors influence learning and its underlying cognitive mechanisms and neural markers.

## 4. Final remarks and future questions

The scope of this review was to look at the state-of-the-art in the neuroscience of human learning as it most naturally occurs, i.e. socially. Social interactions feature as the major catalyst for the human ability to acquire and retain new information [27,42,137], even in robot–human interaction [138]. We have presented evidence showing the crucial role that social interaction plays in human learning across the lifespan. We have argued that the relation between social interaction and learning may be modulated by complex dynamics spanning across behaviour, physiology and the brain [9,139]. As such, we believe that these complex dynamics are unlikely to be fully grasped by experiments that look solely at either the learner or the teacher in isolation, rather than as agents who are part of an interaction. Social agents will inevitably influence and be influenced by each other, and as such the inter-personal dynamics need to be taken into account to fully grasp the cognitive and neural mechanisms subserving social interactive learning.

Taking the cited literature together, a few issues emerge. First, behavioural and cognitive mechanisms involved in learning are mainly studied in isolation: it remains largely unexplored how the experience of learning from others modulates the physiological, behavioural and neural response of the people involved in the interaction, both individually and as a coordinated system. Future studies should integrate the multimodal experimental design and analysis pipeline to grasp the complexity of interactive learning and the mechanisms subserving it.

Second, there are disproportionally more studies on interactive learning in the domain of language acquisition, thus specifically looking at childhood, than any other domain, both in children and adults. Coordination in language use has been demonstrated in adult speakers as well (e.g. [105,106]) due to the tight link between language and social interaction, given the social nature of communication. This coordination may be particularly important in conceptual learning at all ages because of the particular challenges required in learning new concepts. Unlike learning an arbitrary word list, a new concept must be integrated with other existing knowledge of many different types (e.g. [140]). Interactive teaching would allow a learner to try out a new concept and explore how it relates to other concepts with immediate feedback, which is likely to provide richer and more robust learning. Thus, the study of interactive conceptual learning should be integrated with the study of interactive communication, to include coordination in a number of non-verbal behaviours such as gestures and eye-gaze [107,141]. We know very little about how coordination within and across these different channels can support conceptual learning across the lifespan (but see [27]). We also know very little about how we learn in interaction in ways that do not involve communication, for example during large in-person classes where although there is co-presence, there is no or very little active exchange. Studies addressing these questions are needed in order to assess to what extent the benefit of social interaction in learning is content-dependent.

Third, within the domain of interactive learning research, more direct comparisons of observational and interactive social learning are needed. This will be essential to disentangle the contribution of specific factors associated with social contexts that benefit human learning. It is hard to separate individual components because live interaction cannot be easily deconstructed. Future studies using virtual reality might be able to do so by experimentally manipulating which aspects of interaction are most important to learning [142].

Educational neuroscience has recently emerged as a growing discipline worldwide: it aims to identify the key components of naturalistic social engagement during learning. By adopting a data-driven multimodal approach [143], learner–teacher interaction can be dissected to understand information-transfer processes and how these are modulated by interpersonal dynamics [40,144–146]. Here, we have reviewed evidence that allows us to make two claims: first, social interaction is an integral part of human learning. Second, learning in social (and interactive) contexts engages partially different mechanisms from learning in non-social (and non-interactive) contexts. Social interaction in fact employs a series of processes unique to interactive situations, including (but not limited to) joint attention, reciprocity and active attunement, that may be key to support learning in humans.

In conclusion, we presented studies to show the role of social interaction in the first years of life for optimal cognitive and brain development, and demonstrate that social interaction boosts learning in adulthood too. We reported studies that have specifically looked at complex real-world learning (e.g. from object features in children to complex medical curricula in adults) and placed learning in its ecology: these have included social interaction at the different stages of the experimental process to fully grasp the multifaceted mechanisms of interactive learning, and we have discussed their evidence in relation to less comprehensive approaches. Taken together, these suggest that learning via solitary action may not be enough, while learning in *interaction with* others may be a key factor supporting acquisition of new knowledge in the real world.

## Data Availability

This article has no additional data.
